# Predicting age at onset of childhood obesity using regression, Random Forest, Decision Tree, and K-Nearest Neighbour—A case study in Saudi Arabia

**DOI:** 10.1371/journal.pone.0308408

**Published:** 2024-09-26

**Authors:** Salem Hamoud Alanazi, Mali Abdollahian, Laleh Tafakori, kheriah Ahmed Almulaihan, Salman Mutarid ALruwili, Omar Falleh ALenazi

**Affiliations:** 1 School of Science, RMIT University, Melbourne, Victoria, Australia; 2 Department of Mathematics, College of Sciences, Northern Border University, Arar, Saudi Arabia; 3 Maternity and Children Hospital, Arar, Saudi Arabia; 4 North Medical Tower Hospital, Arar, Saudi Arabia; College of Dentistry, Jouf University, SAUDI ARABIA

## Abstract

Childhood and adolescent overweight and obesity are one of the most serious public health challenges of the 21st century. A range of genetic, family, and environmental factors, and health behaviors are associated with childhood obesity. Developing models to predict childhood obesity requires careful examination of how these factors contribute to the emergence of childhood obesity. This paper has employed Multiple Linear Regression (MLR), Random Forest (RF), Decision Tree (DT), and K-Nearest Neighbour (KNN) models to predict the age at the onset of childhood obesity in Saudi Arabia (S.A.) and to identify the significant factors associated with it. De-identified data from Arar and Riyadh regions of S.A. were used to develop the prediction models and to compare their performance using multi-prediction accuracy measures. The average age at the onset of obesity is 10.8 years with no significant difference between boys and girls. The most common age group for onset is (5-15) years. RF model with the *R*^2^ = 0.98, the root mean square error = 0.44, and mean absolute error = 0.28 outperformed other models followed by MLR, DT, and KNN. The age at the onset of obesity was linked to several demographic, medical, and lifestyle factors including height and weight, parents’ education level and income, consanguineous marriage, family history, autism, gestational age, nutrition in the first 6 months, birth weight, sleep hours, and lack of physical activities. The results can assist in reducing the childhood obesity epidemic in Saudi Arabia by identifying and managing high-risk individuals and providing better preventive care. Furthermore, the study findings can assist in predicting and preventing childhood obesity in other populations.

## Introduction

The global prevalence of obesity among children and adolescents aged 5-19 has increased ten-fold over the past four decades [1] and has become one of the most serious public health challenges of the 21st century [2]. As of 2021, the latest available data shows that an estimated 340 million children and adolescents aged 5-19 years were overweight or obese worldwide.

Childhood overweight and obesity increase the risk of non-communicable diseases such as diabetes and cardiovascular disease in adulthood. This highlights an urgent need for an understanding of the development of childhood obesity to facilitate improved monitoring to reduce the complications of delayed diagnosis.

The study conducted by [3] investigates the connection between hypertension and childhood obesity. The paper highlights the crucial need for early identification and management of hypertension as a preventive measure against long-term health complications. Childhood obesity is caused by a lack of physical activity, unhealthy eating habits, and genetic factors and can lead to different non-communicable diseases. [4].

The patterns in childhood obesity rates in the United States (U.S.) spanning the period from 1999 to 2018 were analysed by [5]. The study found that the incidence of obesity among children aged 2 to 4 years decreased by 31.8% between 2010-2011 and 2016-2017, while there was no significant change among children aged 5 to 11 years during the same period. The authors suggest that targeted interventions during early childhood may have contributed to the observed decrease in obesity incidence among the younger age group. In comparison, further research is needed to understand the lack of change in the older age group. The prevalence of childhood obesity and severe obesity in the U.S. between 1999 and 2016 had a significant rise [6]. Approximately one in eight children aged 3-5 years were obese, and one in fifty were extremely obese [7].

The prevalence of obesity among children aged 6 to 11 years in Sharjah, UAE was 17.2%, with a higher impact on male children compared to females [8]. The ABIS study on children aged 2.5 to 8 years in Sweden has identified parental obesity, high birth weight, and low physical activity as significant predictors [9]. In Sohag, Egypt 16.2% of children aged 6 to 12 years were overweight and obese [10]. The patterns of change in adiposity measures over time in a population of low-income Hispanic children showed a distinct change in adiposity measures, with some children showing a steady increase in body mass index (BMI) while others demonstrated a more rapid increase in BMI [11]. The study highlights the importance of early intervention to prevent obesity in low-income Hispanic children and recommends tailored interventions based on the trajectory of adiposity measures in individual children.

The studies [12–15] present a comprehensive examination of childhood obesity, encompassing its etiology, associated comorbidities, and available treatment modalities. The authors underscore the substantial global rise in childhood obesity prevalence and delineate a spectrum of factors contributing to its onset, encompassing genetic, environmental, and behavioral influences. Additionally, the studies explore the comorbidities correlated with childhood obesity, including cardiovascular disease, type 2 diabetes, and sleep apnea. Ultimately, the authors provide an overview of treatment options for childhood obesity. A Denmark study showed that a primary prevention intervention targeting obesity-susceptible, healthy-weight preschool children effectively mitigated fat and weight gain [16]. Similarly, research conducted [17] in the U.S. revealed an association between patterns of early life BMI and the prevalence of overweight and obesity in 8-year-old children. A German study showed that birth weight and weight changes during infancy to early childhood can predict BMI in adolescence [18]. This longitudinal study, spanning from birth to age 14, established that early weight gain significantly predicts adolescent BMI.

These findings propose that trajectories of BMI in early life could serve as valuable indicators for identifying children at risk of obesity, thereby aiding in the formulation of informed prevention strategies.

A study on 7-year-old European children in Auckland, New Zealand identified maternal smoking during pregnancy, elevated maternal pre-pregnancy BMI, and diminished levels of physical activity as key factors associated with childhood obesity [19]. A U.S. study on associations between the age of onset of overweight/obesity and children’s socio-demographic characteristics revealed a significant link between the frequency of moving houses and an earlier onset of obesity in children, with an approximate advancement of 4 months. In Norway, a noteworthy escalation in weight-for-height and skinfold thickness has been observed among children over recent decades [20]. Studies in Ghana, [21] and Bahir Dar City, Ethiopia, [22] identified low participation in sports activities, maternal tertiary education, higher household socioeconomic status, and attending private school as significant factors associated with childhood obesity. In China, eating speed, sleep duration, birth weight, paternal BMI, frequency of fast-food intake, gestational weight gain (GWG), and maternal pre-pregnancy BMI were associated with childhood obesity [23]. The analysis of BMI data from 5173 children aged 3 and 5 showed that children born large for gestational age, overweight/ obese at age 3 often face an increased risk of obesity at age 5 [24].

Research on large pediatric health data in the U.S. showed age is a significant predictor of obesity, and females with lower income have a higher obesity risk [25].

Analysis of the National Health and Nutrition Examination Survey from 2001–2008 in the U.S. showed that the prevalence of obesity was higher among older children (aged 12-19) than younger children (aged 2-11) with the highest prevalence among non-Hispanic black adolescents (aged 12-19) [26]. Similar studies have used machine learning [27, 28] to predict childhood and adolescent obesity and identified age as a key factor in predicting childhood and adolescent obesity with older age groups having higher rates [29, 30].

### Obesity and overweight in Saudi Arabia

The prevalence of childhood and adolescent obesity in Saudi Arabia has been steadily increasing in recent years [31, 32]. A cross-sectional study involving 20,000 children in the Eastern Province showed that the prevalence of obesity was higher among boys (16.2%) than girls (12.7%) and increased with age [31]. The highest prevalence was observed among children aged 10-12 years and adolescents aged 15 to 19 years. The significant predictors of childhood obesity were Parents’ education level, age, glucose level, and blood pressure. It has been reported that the overall prevalence of overweight and obesity among children and adolescents in the country is 36.5%, with 17.9% categorized as overweight and 18.6% as obese [33]. The study also showed a higher prevalence among boys (41.3%) compared to girls (31.6%). Various risk factors, including a family history of obesity, insufficient physical activity, and high consumption of fast food and sugary drinks have been associated with childhood obesity [34].

Investigating the link between physical activity, sedentary behaviors, breakfast consumption, and BMI among Saudi students aged 10-15 years, [35] discovered that sedentary behaviors, such as prolonged screen time and lack of physical activity, correlated positively with higher BMI. Conversely, regular breakfast consumption was associated with a lower BMI. In a review, addressing the unique challenge of obesity in Saudi Arabia, [36] highlighted the nation’s dual status of high-income and a developing country. Saudi Arabia with an overall obesity rate of 33.7% is ranked 15th globally [36]. It is projected that, by 2017, the prevalence would reach 38.2% among men and 67.5% among women, resulting in an overall rate of 52.9%. The rates were projected to reach 41.4%, 77.6%, and 59.5%, respectively.

The research conducted in 2020 showed that the percentage of obesity and overweight among children aged 6 to 14 years old in Majmaah, Saudi Arabia was 10.1% and 18.9% respectively. Male children had an obesity rate of 11.2% and an overweight rate of 17.6%, while female children had an obesity rate of 9.2% and an overweight rate of 19.8% [37]. A primary-school-based survey was used for these estimations. The overall prevalence of overweight and obesity among male children aged 7 to 15 years in Al-Ahsa, was 29.6% of which 10.8% were overweight, 3.8% obese, and 15% extremely obese. The rates have been increasing with increasing age. The high prevalence of obesity was linked to early childhood obesity, parental obesity, mother’s employment, family income, the number of snacks and fast food eaten, physical inactivity, and time spent viewing television. Additionally, emotional eating, family meals, and consistent meal times were independently associated as well [38].

Predicting the age at the onset of obesity would enable health professionals to develop an early intervention plan involving lifestyle changes and dietary advice for children at risk of developing obesity. Early detection and interventions will also reduce the financial burden of treating obesity later in life. Furthermore, understanding the factors associated with age at the onset of childhood obesity would help to develop informed and effective public health policies and strategies to reduce childhood obesity and improve the quality of life for those affected. Various machine learning algorithms have been used to predict age and to identify the risk factors associated with childhood obesity [28, 39].

## Motivation and the objective of the proposed research

Childhood and adolescent obesity is a major problem around the world [24] and has significantly increased in the Middle East in recent years. Among Saudi Arabian children, the prevalence of obesity is rapidly increasing and has become a critical public health concern [31, 33, 40]. Obesity in childhood can lead to various health problems in adulthood, including diabetes, heart disease, and certain cancers.

Despite efforts to reverse this situation, the prevalence remains high, suggesting that the current intervention approach is inadequate [1]. The existing obesity research conducted in Saudi Arabia has examined the different aspects of childhood and adolescent obesity but has not modelled the age at the onset of childhood obesity. This study aims to fill the gap by utilising a secondary data source, to develop a reliable prediction model to estimate the age at the onset of obesity for children and adolescents aged 3-19 years old and to identify the potential environmental and clinical risk factors associated with the age. The performance of the predictive models MLR, RF, DT, and KNN will be compared using a variety of metrics such as the coefficient of determination *R*^2^, the root means squared error (RMSE), and the mean absolute error (MAE). The results of this study will provide insights into the potential of machine learning algorithms to predict the age at the onset of childhood obesity in Saudi Arabia. To the best of our knowledge, no previous studies have modelled the age at the onset of childhood obesity in Saudi Arabia. This research can facilitate early diagnosis and effective preventive strategies to reduce/prevent childhood and adolescent obesity and its related health problems, such as cardiovascular diseases, diabetes, and psychological disorders. The outcomes of this research both support the improvement of the nation’s health and add to the current research on childhood obesity in diverse populations while recognising the lack of obesity studies for Saudi Arabia children. The model can facilitate early diagnosis and intervention plans to reduce/prevent childhood obesity. The results revealed that R.F. outperforms other models followed by MLR, DT, and KNN. The results also show that the age at the onset of obesity was linked to several demographic, medical, and lifestyle factors including gender, education and income level of parents, first-degree family history of obesity, autism, gestational age, height, nutrition in the first 6-months, weight at birth, child sleep hours, consanguineous marriage and lack of Physical activities.

## Data analysis and model development

This section outlines the data collection, model development and evaluation. The statistical software R was used to perform the analysis.

## Data collection

De-identified data for this research has been collected from hospitals in the Arar and Riyadh regions of Saudi Arabia between 2011 and 2021. A total of 300 patient records from 2011-2021 have been extracted for children aged 3-19 years. A range of available medical, demographic, and lifestyle variables that were identified by other researchers as being significant factors for childhood obesity were selected. These variables are listed in Table 1. Ethical approval was obtained from the RMIT University Human Research Ethics Committee in Australia and the Research Ethics Committee of the Ministry of Health in Saudi Arabia. The need for informed consent was waived by the ethics committee as this was a retrospective study of medical records.

**Table 1:** List of variables used for this study.

	Demographic characteristics		Child characteristics
1	Gender	1	Sleep Hours regular or irregular
2	Residency	2	No of times consuming soft drinks per day
3	consanguineous marriage	3	No of times consuming sweet and chocolate per day
4	Income status	4	Exercise for 60 minutes per week
5	Education level of parent	5	No of hours watching TV per day
	**Medical history**	6	No of times eating fast food per week
1	Age of child at diagnosis	7	Eating while watching TV (yes or no)
2	High and weight at diagnosis		**Obstetric history**
3	BMI at diagnosis	1	Birth delivery mode
4	Is there in the family anyone with obesity?	2	natural (SVD) or caesarean section (CS)
5	Second-degree relative	3	Gestational age (in weeks)
6	First-degree relative: Father—mother—siblings	4	Weight at birth (Kg)
7	autism		
8	Diabetes		
9	Nutrition history in the first 6 months: (Breastfeeding -Introduction to Cow’s milk—Both)		
10	When did mother start to introduce solid food? (<6 months or >6 months)		

**Fig 1:**
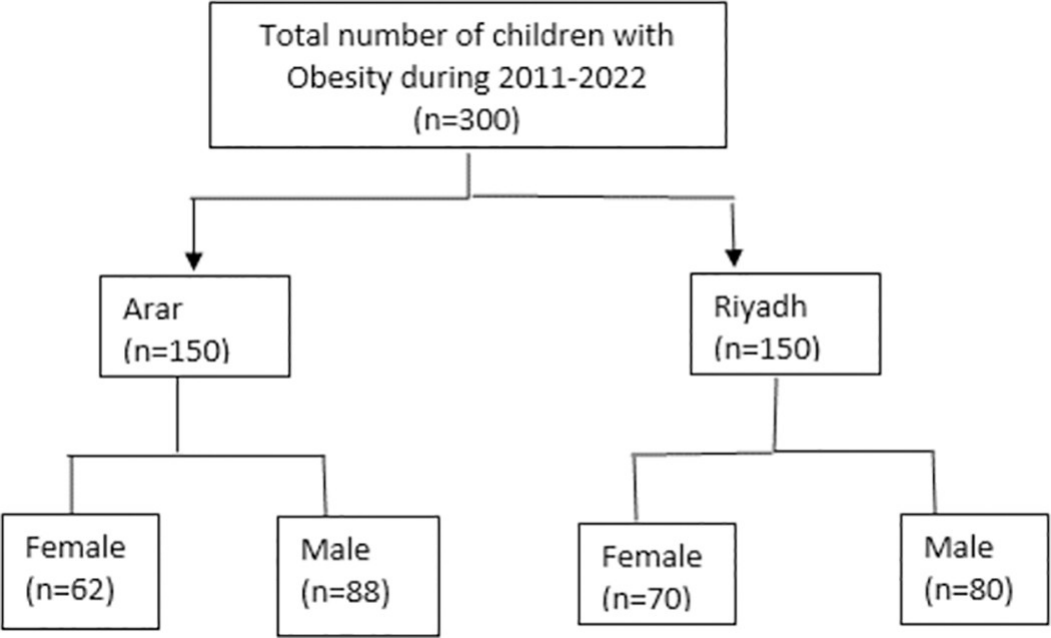
Sample size by city and gender.

The number of males and females in each city is shown in Fig 1.

## Descriptive statistics, t-test and ANOVA

Descriptive statistics including summary statistics and frequency counts have been used to describe the data. The statistics reported include mean, median, standard deviation, minimum and maximum values. Graphs have also been used to investigate the trend of age at the onset of obesity and to extract information on the distribution of age at the onset of obesity. Furthermore, a t-test was employed to assess the disparity in the age at the onset of obesity between different genders. Additionally, the analysis of variance (ANOVA) was utilized to examine the variation in the age at the onset of obesity across genders and cities.

## Models

**Fig 2:**
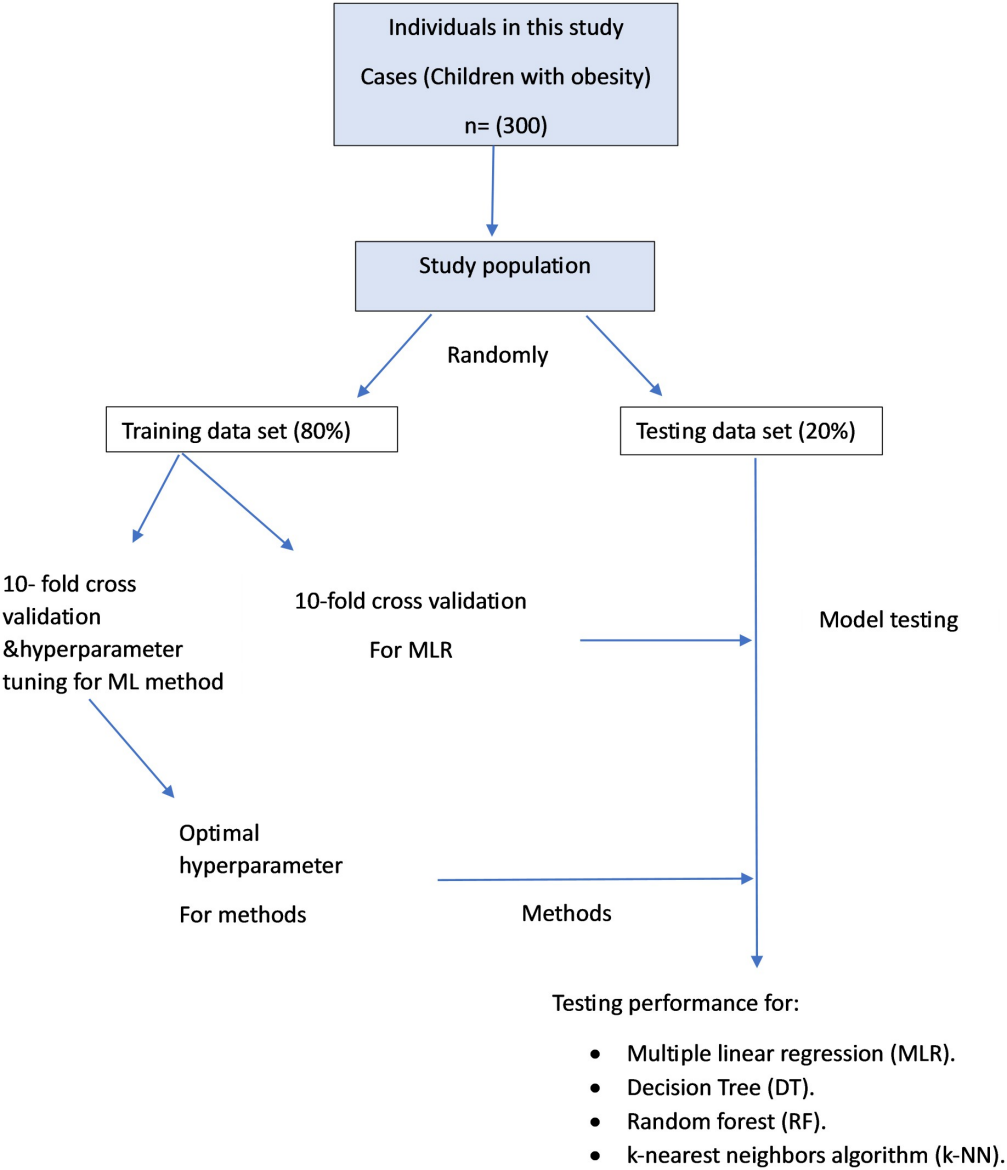
Flowchart of models performance.

The modelling techniques used to predict the age at the onset of childhood obesity include MLR, RF, DT, and KNN Fig 2. The dependent variable is the age at the onset of obesity. All variables listed in Table 1 (except age) have been used as independent variables. The assumptions of the various models have also been tested. The data were randomly split into a training (80%) and testing set (20%). The developed models were tested as shown in Table 8. The best models have been selected based on the highest coefficient of determination *R*^2^, the smallest root mean squared error(RMSE), and the smallest mean absolute error (MAE).

## Multi linear regression model

Multi linear regression is a statistical technique used to identify relationships between several independent variables and a dependent variable. The model is a linear equation of the form

$$\begin{array}{c} Y=\beta_{0}+\beta_{1}X_{1}+\beta_{2}X_{2}+\cdots +\beta_{n}X_{n}, \end{array}$$ (1)

where *Y* is the dependent variable, *X*_1_, *X*_2_, …, *X*_*n*_ are the independent variables, and *β*_0_, *β*_1_, *β*_2_, …, *β*_*n*_ are the coefficients. The coefficients are estimated by fitting a linear regression model to a set of observed values. This technique has been widely used in the health area to investigate the relationship between a variety of health-related variables [41].

## Decision Tree model

Decision tree model uses a tree-like structure to map out the possible outcomes of a situation or decision. It is typically used to illustrate the relationship between the different variables involved in a decision, or to represent the potential outcomes of a decision. The tree is composed of nodes, branches, and leaves. The branches can be split into a variety of different conditions or questions. DT models have been used in health modelling by many researchers [42–44]. They are used to predict the outcomes of different scenarios. For example, DT can be used to identify patterns in data that can lead to more accurate predictions of the outcomes of treatments; they can also be used to make decisions about the best course of treatment for a particular patient; and/or can be used to analyse the effect of different factors on the likelihood of a successful outcome and identify the best treatment based on these factors.

## Random Forest model

Random Forest models are supervised machine learning methods used for both classification and regression problems. In an RF model, Multiple DT models are combined to form a single predictive model. The individual Decision Trees are grown using a random subset of the data, and the output of the trees is combined to create a final prediction. RF models are used in health areas to identify complex non-linear relationships between variables [43–45]. The Decision Trees in an RF model can capture multiple interactions between variables, allowing for more accurate predictions than a single DT. Additionally, the random subset of data used to grow each tree helps to reduce overfitting (common when using a single DT). The importance score assigned to each variable in RF is used to identify the most important prediction variables. This information can be used to better understand the underlying process and to create more accurate predictions. Finally, RF models are relatively easy to use and can produce high-quality results with minimal tuning. This makes them an attractive choice for health modelling tasks.

## K-Nearest Neighbour model

The k-Nearest Neighbour model is based on the concept of instance-based learning, or memory-based learning. In the KNN model, data points are classified according to the values of the K-Nearest Neighbours. KNN models are simple to understand and can be used for both classification and regression tasks and are useful in health modelling [46]. They can identify patterns in data that may not be easily detected by other types of models [47]. KNN models often are used to predict the risk of a particular disease or health condition based on the features of a patient’s data, for example, the risk of developing diabetes based on the patient’s age, gender, lifestyle, and other factors.

## Descriptive statistics results

**Fig 3:**
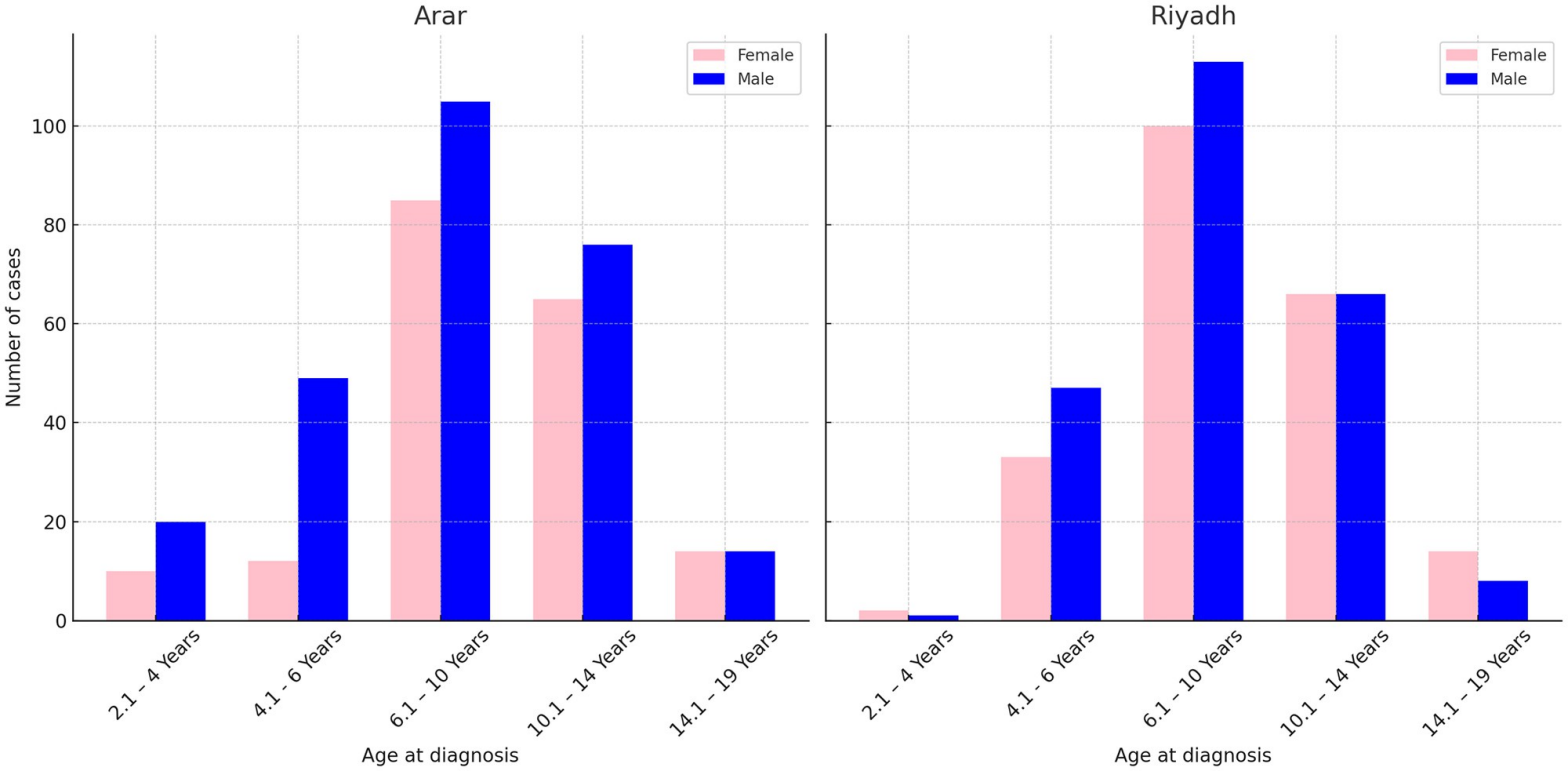
Overall distribution of age with gender and cities.

**Fig 4:**
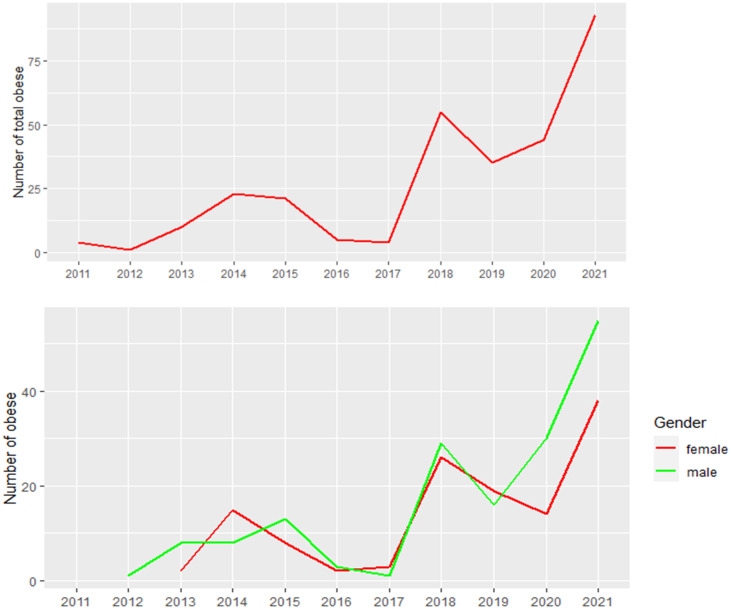
Trend of overall cases and cases by gender between 2011 and 2021.

**Fig 5:**
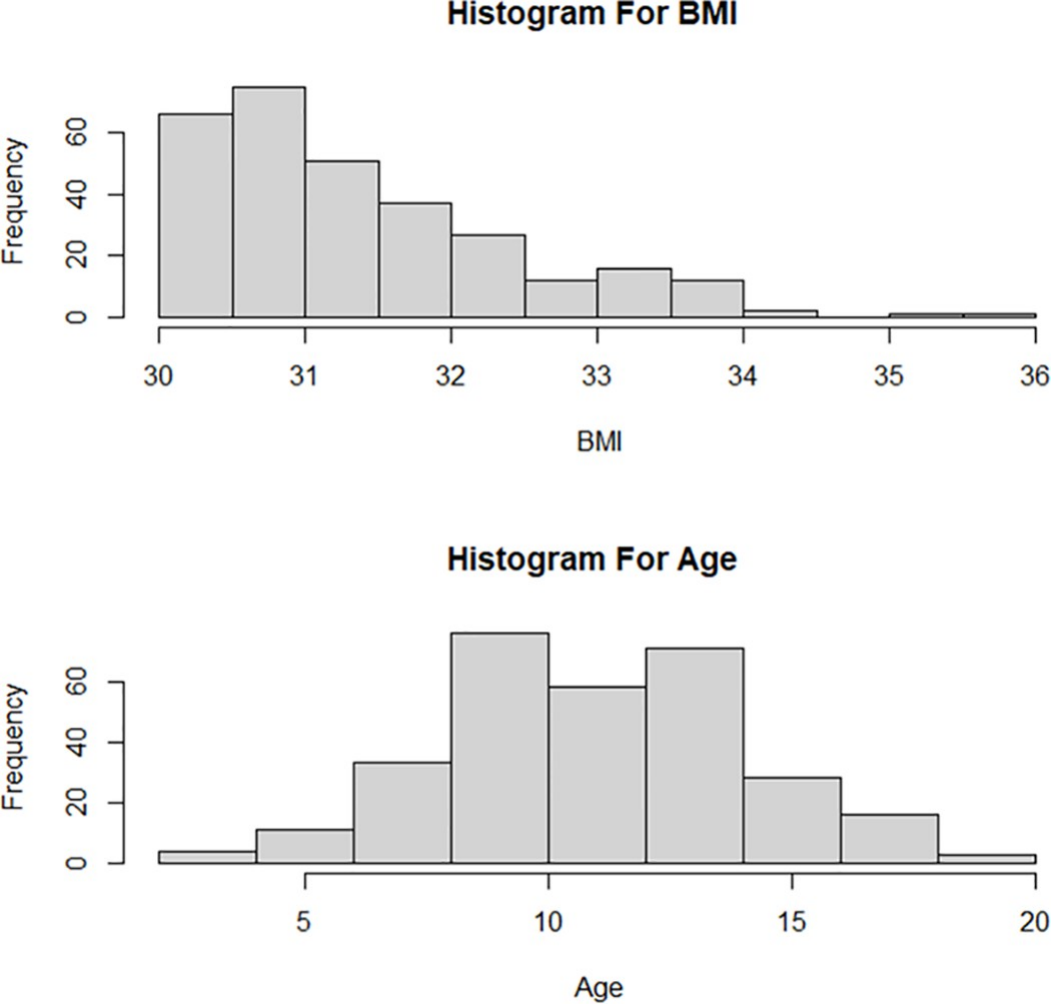
BMI and age at diagnosis.

**Fig 6:**
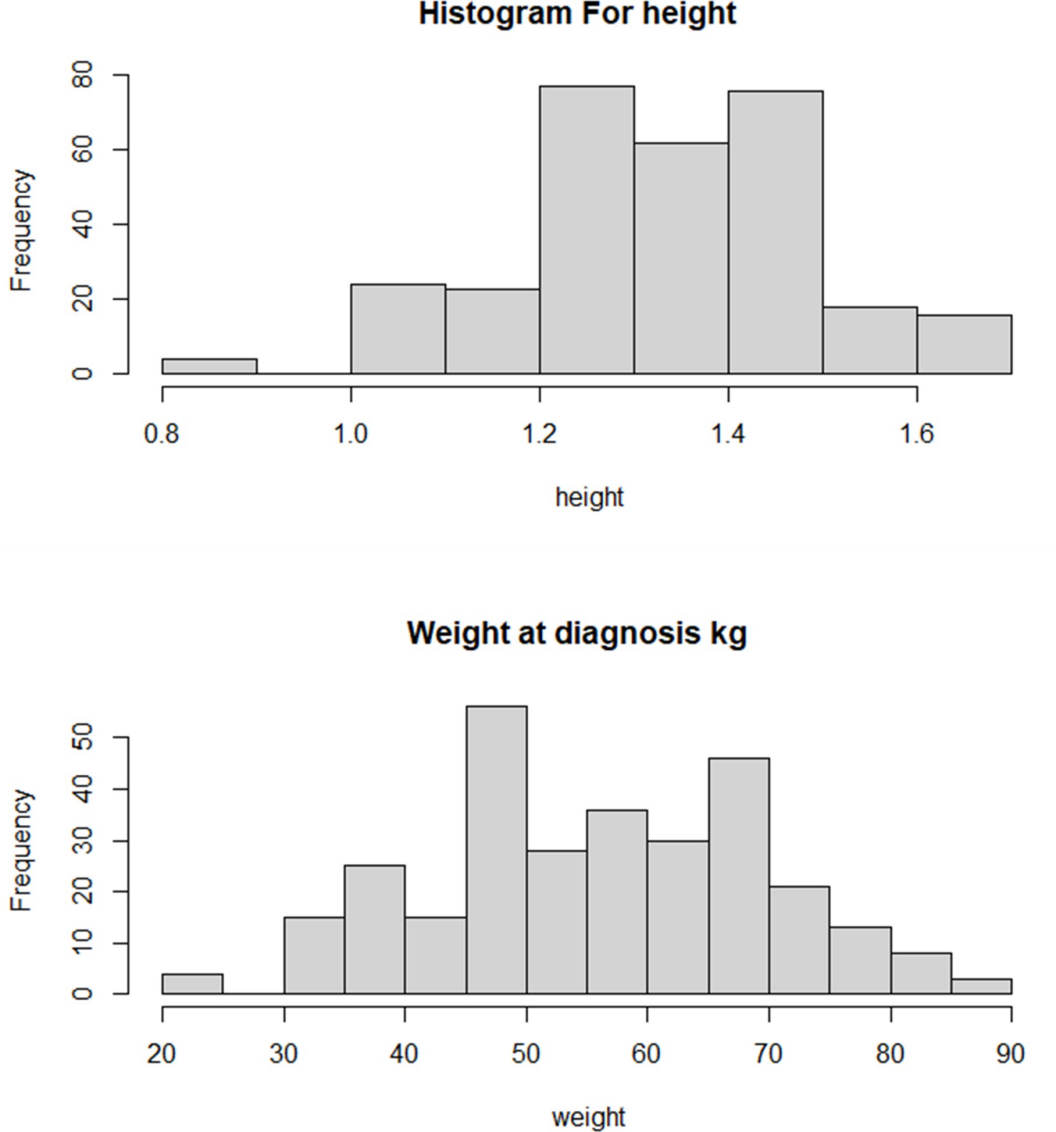
Height and weight at diagnosis.

The distribution for age by gender and cities is presented in Fig 3. Arar and Riyadh’s most common age group is 6.1-10 years. Overall, there are more males with obesity than females in both cities. The trend of the recorded cases between 2011 and 2021 is shown in Fig 4. Additionally, as shown in Fig 4, males had a greater prevalence of obesity than females during this period. The descriptive statistics for the BMI and age by gender are provided in Table 2 and Fig 5. The overall mean age is 10.84 (SD = 3.12). When stratified by gender, the mean age for males (n = 168) was 10.79 (SD = 2.75), and for females (n = 132) was 10.89 (SD = 3.54). The overall BMI mean is 31.38 (SD = 1.06), for males (n = 168) was 31.30 (SD = 1.05), and for females (n = 132), was 31.48 (SD = 1.07). Fig 5 displays a positively skewed distribution of the BMI, with 50% of cases falling between 30.63 to 31.97 (IQR = 1.34). The distribution of the age at diagnosis is approximately normal, with most children falling between 5 to 15 years of age. The descriptive statistics for the height and weight are shown in Table 3 and Fig 6. The mean weight is 56.62 kgs (SD = 13.71), and the mean height is 1.33 meters (SD = 0.16). The mean weight, height, and BMI are similar for males and females. The frequency counts for the categorical variables broken down by demographic, Socioeconomic, genetic, and nutritional history categories are shown in Table 4. The outputs of the t-test show that there are no significant differences between the age at the onset of obesity between males and females (p = 0.79), Table 5. The results of the ANOVA show significant differences between the main effect of cities on age at the onset of obesity (p =.04); the age at the onset of obesity is significantly higher in Arar compared to Riyadh, Table 6.

**Table 2:** Descriptive statistics for BMI and age.

	BMI			Age		
	All	Female	Male	All	Female	Male
N	300	132	168	300	132	168
Mean	31.38	31.48	31.30	10.84	10.89	10.79
Std.Deviation	1.06	1.07	1.05	3.12	3.54	2.75
Median	31.09	31.16	31.03	10.60	11.50	10.35
Minimum	30.01	30.01	30.01	3.20	3.20	4.30
lower quarter	30.63	30.73	30.47	8.78	8.40	9.20
upper quarter	31.97	32.00	31.83	13.12	32.00	12.43
Maximum	35.58	35.58	34.23	18.60	18.60	18.20

**Table 3:** Descriptive statistics for height and weight.

	Height			Weight		
	All	Female	Male	All	Female	Male
N	300	132	168	300	132	168
Mean	1.33	1.34	1.33	56.62	57.39	56.02
Std.Deviation	0.16	0.18	0.14	13.71	15.49	12.15
Median	1.37	1.38	1.31	58.00	61.50	54.50
Minimum	0.87	0.87	1.01	23.00	23.00	32.00
lower quarter	1.24	1.21	1.25	47.00	45.00	48.00
upper quarter	1.44	1.45	1.42	68.00	69.00	64.00
Maximum	1.70	1.70	1.69	88.00	88.00	87.00

**Table 4:** Frequency count percentage for categorical variables.

Variables	Cases (%)
Demographic &Socioeconomic	
City: Arar	150 (50.0)
Riyadh	150 (50.0)
Gender: Female	132 (44.0)
male	168 (56.0)
Residency: Rural	81 (27.0)
Urban	219 (73.0)
Income status: High	55 (18.33)
Low	15 (5.00)
Middle	230 (73.67)
Education level of parent: Father	155 (51.67)
non-university	
university	145 (48.33)
Mother non-university	175 (58.33)
university	124 (41.33)
Medical history(genetic)	
consanguineous marriage: NO	174 (58.0)
YES	126 (42)
Family history of obesity: Yes	295 (98.33)
No	5 (1.67)
First-degree of obesity: Father	83 (27.67)
Mother	94 (31.33)
Siblings	15 (5.0)
None	108(36.0)
Second-degree relative: NO	191 (63.67)
Yes	109 (63.33)
Autism: NO	223 (77.67)
YES	77 (25.67)
Diabetes: NO	296 (98.67)
Yes	4 (1.33)
Nutritional history	
Nutritional history: Breast feeding	30 (10.0)
Introduction to cow’s Milk	111 (37.0)
Both	159 (53.0)
Introducing to solid food:(<6 months)	46 (15.33)
(>6 months)	254 (84.67)

**Table 5:** T-test results comparing age at onset of obesity between males and females.

Gender Level	N	Mean	CI	t	df	p-value
Male	132	10.79	(-0.64, 0.83)	0.25	242.06	0.79
Fmale	168	10.88				

**Table 6:** ANOVA results comparing age at onset of obesity between gender and cities.

Variables		Mean Difference	CI	P-value
Gender	Male-Female	-0.09	(-0.80, 0.61)	0.79
Cities	Riyadh-Arar	-0.73	(-1.43, -0.02)	0.04
Gender: City	Male: Arar- Female: Arar	-0.15	(-1.48, 1.17)	0.99
	Female: Riyadh -Female: Arar	-0.75	(-2.15, 0.64)	0.50
	Male: Riyadh-Female: Arar	-0.86	(-2.22, 0.48)	0.35
	Female: Riyadh-Male: Arar	-0.59	(-1.88, 0.68)	0.62
	Male: Riyadh-Male: Arar	-0.71	(-1.95, 0.52)	0.44
	Male: Riyadh-Female: Riyadh	-0.11	(-1.43, 1.19)	0.99

## Modelling results

This section presents the modelling results of MLR, DT, RF, and KNN. The summary comparison is presented in Table 8, and Fig 9.

## Multi linear regression model

We have developed MLR models to predict age at the onset (y) based on all variables listed in Table 1 (except age). The analysis of the variables that influence the age at the onset of obesity together with their corresponding P-value and 95% confidence interval is provided for in Table 7. Results presented in Table 8 display an *R*^2^ of 0.98, RMSE of 0.46, and MAE of 0.37 for the testing data. The plot of training and testing data for the model is shown in Fig 8.

**Table 7:** Significant variables of the MLR model.

Variables	*β*	95%CI	p-value
Intercept	11.03	(10.10, 11.96)	<2e-16 ***
Education level of Father University	0.17	(-0.06, 0.40)	0.14
autism Yes	-0.31	(-0.57, -0.05)	0.01 *
Gestational Age weeks	-0.09	(-0.19, 0.01)	0.08
Child sleep hours Regular	-0.25	(-0.49, -0.005)	0.04 *
Watch TV per day	-0.13	(-0.31, 0.05)	0.16
Hight	2.33	(1.62, 3.04)	5.83e-10 ***
weight	0.57	(-0.14, 1.28)	0.12

***Significant at p-value < 0.001,

**Significant at p-value < 0.01,

*Significant at p-value < 0.05,

. Significant at p-value < 0.1

**Table 8:** Comparison table for all the models.

model	Training data			Testing data		
	*R* ^2^	RMSE	MAE	*R* ^2^	RMSE	MAE
MLR	0.945	0.700	0.377	0.983	0.457	0.370
Random forest	0.960	0.602	0.244	0.984	0.442	0.284
Decision Tree	0.944	0.712	0.374	0.974	0.562	0.416
KNN	0.737	1.786	1.396	0.746	2.281	1.708

## Decision Tree model

**Fig 7:**
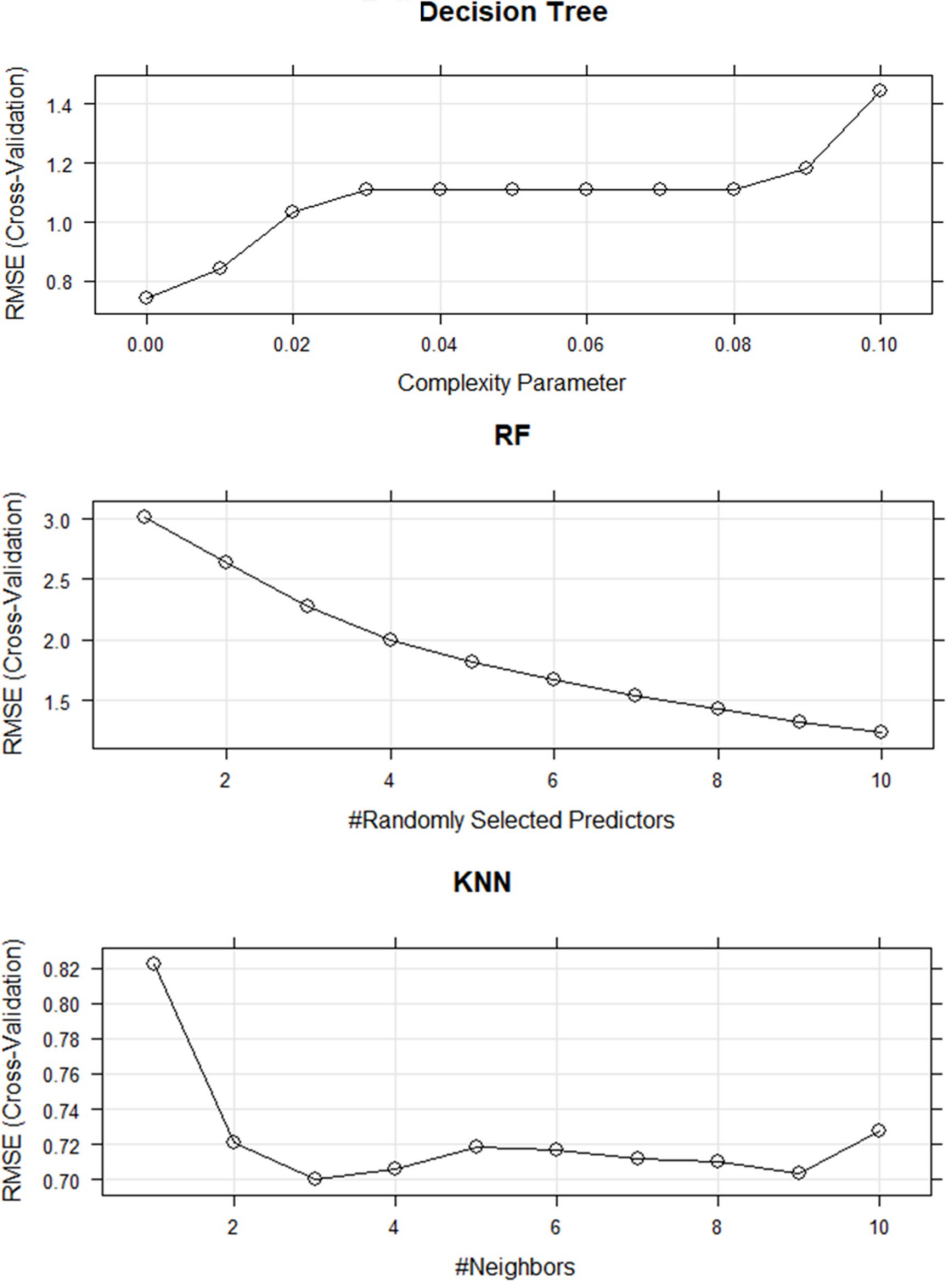
Tuning hyperparameter for RF, KNN, and DT models.

**Fig 8:**
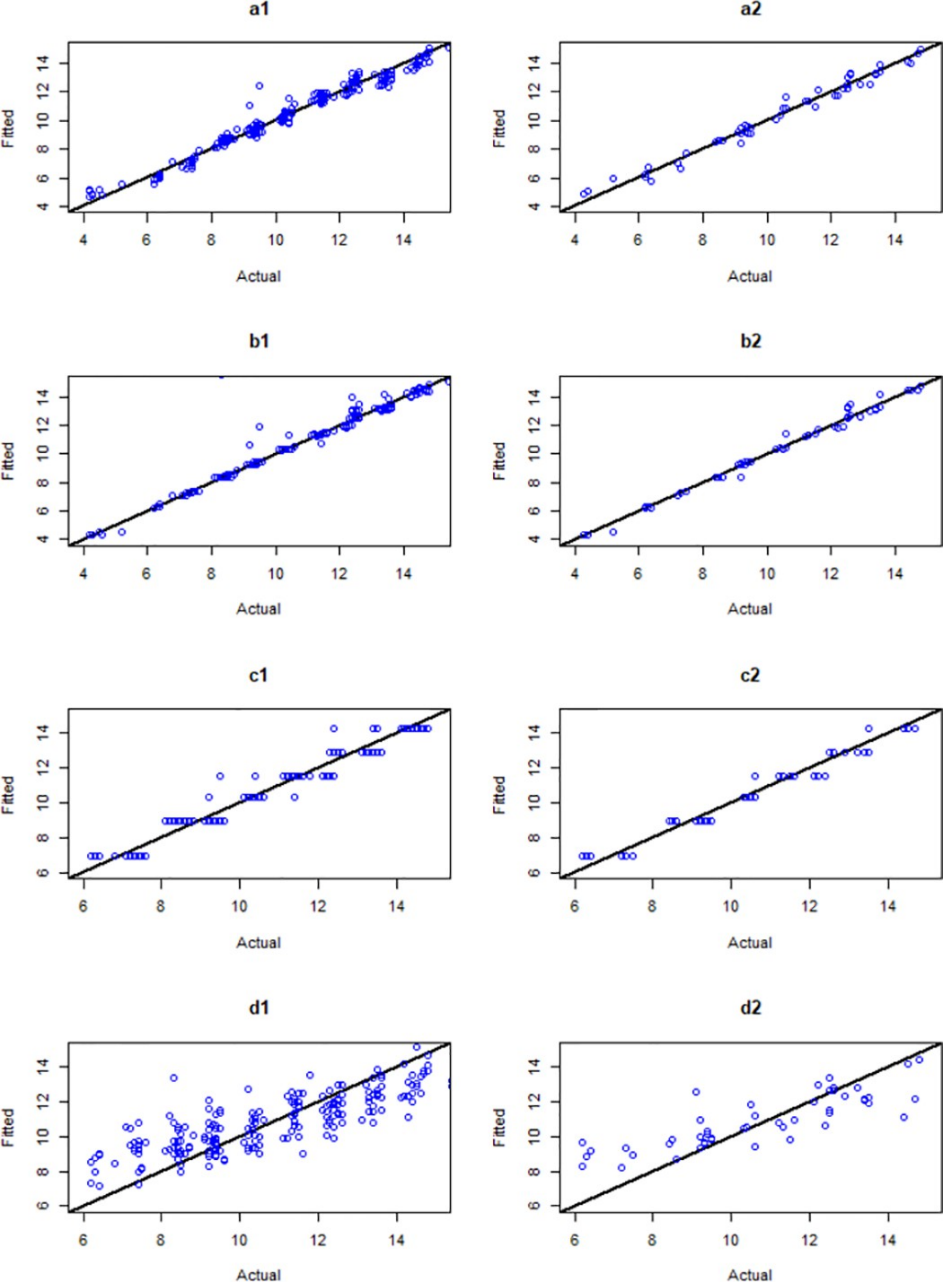
Plots of training and testing data for the models. (a1) MLR for the training data and (a2) testing data, (b1) RF for the training data and (b2) testing data, (c1) DT for the training data and (c2) testing data, (d1) KNN for the training data and (d2) testing data.

DT models have been built to predict age at the onset (y) using all variables listed in Table 1 (except age). Moreover, the hyperparameter governing the randomization of the split variable feature in Decision Trees which is commonly denoted as the “cp” parameter has been considered to determine the tree’s complexity. Through 10-fold cross-validation based on the training data, we observed that the best (cp) for the DT is 0.005 as shown in Fig 7. The evaluation results of the model on the testing data show an *R*^2^ value of 0.97, an RMSE of 0.56, and an MAE of 0.42, Table 8. The plot of training and testing data for the model is shown in Fig 8.

## Random Forest model

RF models have been built to predict the age at the onset (y) using all independent variables. The hyperparameter that controls the split variable randomization feature of RF is often referred to as mtry. This is the number of variables randomly sampled as candidates at each split and helps to balance the trade-off between a low correlation and reasonable strength. Through 10-fold cross-validation based on the training data, we observed that the best mtry for the RF model is 10, Fig 7. Table 8 displays an (*R*^2^ = 0.98, RMSE = 0.44, and MAE = 0.28 for the model. The plot of training and testing data is shown in Fig 8.

## K-Nearest Neighbour model

The K-Nearest Neighbour models have been developed to predict the age at the onset of childhood obesity. The hyperparameter that controls the split variable randomization feature of the KNN is referred to as the (K) parameter. It controls the number of variables in the model. Through 10-fold cross-validation based on the training data, we observed that the best (K) for the KNN is 3, Fig 7. The evaluation metrics for the testing data are *R*^2^ = 0.75, RMSE = 2.28, and MAE = 1.71, Table 8. The plot of training and testing data for the model is shown in Fig 8.

## Model validation

**Fig 9:**
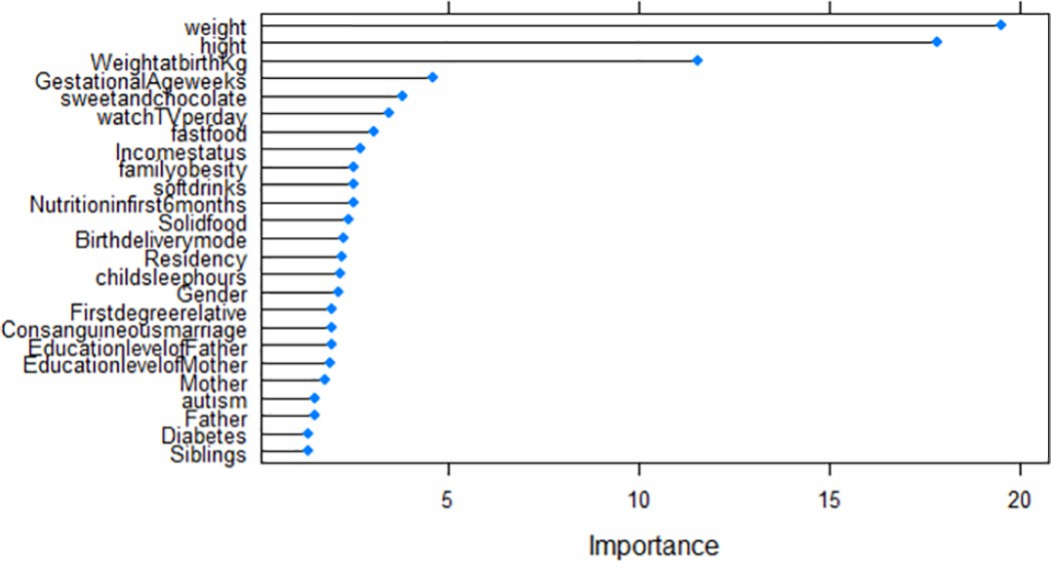
DT importance variables.

**Fig 10:**
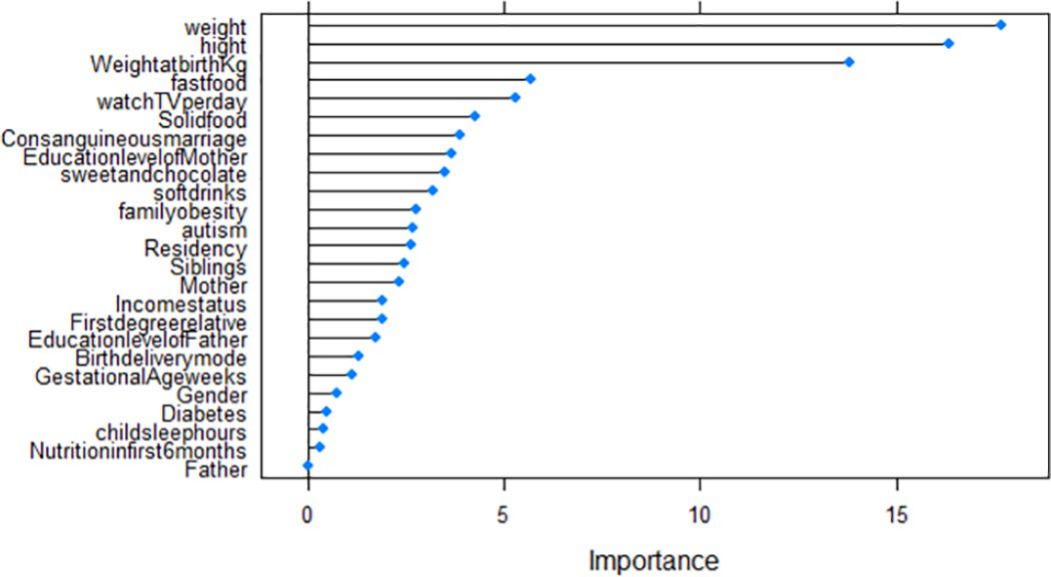
RF importance variables.

**Fig 11:**
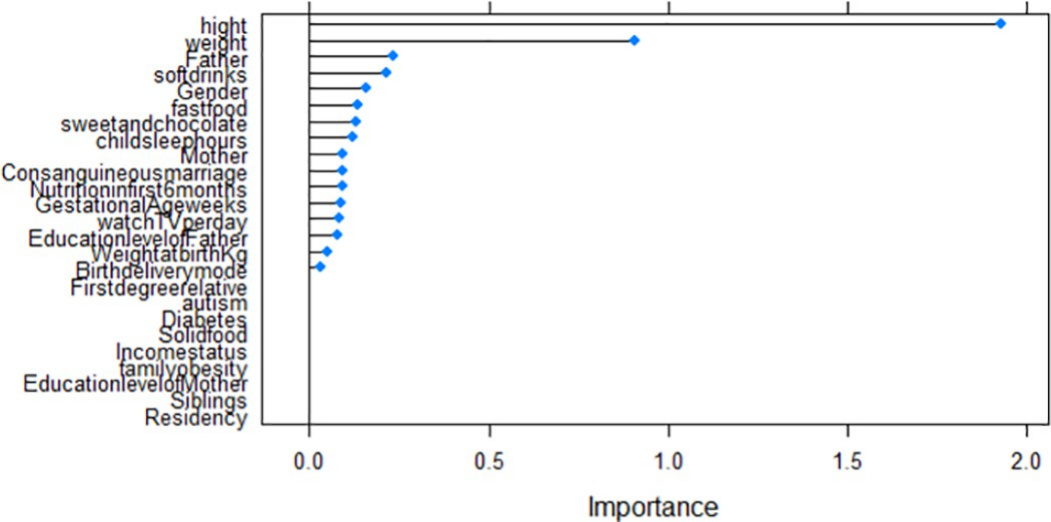
KNN importance variables.

Validation of models was conducted using their corresponding *R*^2^, RMSE, and MAE of the test data set. The results summarized in Table 8 clearly show that RF and MLR with *R*^2^ = (0.983 and 0.984), RMSE = (0.45 and 0.44) and MAE = (0.37 and 0.28) respectively outperform the DT model. Based on the highest *R*^2^, the smallest RMSE and MAE in the testing data, the RF model was the best-performing model followed by MLR, DT, and KNN. The significant predictors of the age at the onset of obesity for each of the models are summarised in Table 9 and Fig 9 (DT), Fig 10 (RF), and Fig 11 (KNN).

**Table 9:** Significant variables for all the models.

Model	Significant variables
MLR	Hight
	Autism
	Child sleep hours Regular
DT	Weight.
	Height.
	Weight at birth Kg.
	Gestational age (in weeks).
	Sweet chocolate per day.
	Watch TV per day.
	Fast food.
	Income status.
	Family history of obesity.
	Soft drink.
	Nutrition in first 6 months.
RF	weight.
	Hight.
	Weight at birth Kg.
	Fast food.
	Watch TV per day.
	Solid food.
	Consanguineous marriage.
	Education level of mother.
	sweet chocolate per day.
	Soft drink.
	Family history of obesity.
KNN	Hight.
	weight.
	Family history of obesity.
	Soft drink.
	Gender.
	Fast food.
	sweet chocolate per day.
	Child sleep hours
	Consanguineous marriage.
	Nutrition in the first 6 months.
	Gestational age (in weeks).
	Watch TV per day.
	Education level of father.
	Weight at birth Kg.

## Discussion

The increasing rate of childhood and adolescents obesity is a significant concern in Saudi Arabia. Predicting the age at which children/ adolescents might become obese and identifying the early life factors that are associated with the development of childhood obesity would help medical practitioners with early diagnosis and treatment. In this paper, Multi linear regression, Random Forest, Decision Tree, and K-Nearest Neighbour algorithms were used to predict the age at the onset of childhood obesity in Saudi Arabia and to identify the significant factors associated with it. De-identified data from 2011 to 2021 collected from hospitals in the Arar and Riyadh regions were used for the analysis. The efficacy of the models was assessed and compared using accuracy measures such as *R*^2^, RMSE, and MAE for the testing data set. The results show that RF (*R*^2^ = 0.98, RMSE = 0.44, and MAE = 0.28) outperforms other models followed by MLR (*R*^2^ = 0.98, RMSE = 0.46, and MAE = 0.37), DT (*R*^2^ = 0.97, RMSE = 0.56, and MAE = 0.42), and KNN (*R*^2^ = 0.75, RMSE = 2.28, and MAE = 1.71).

RF combines multiple decision trees, which reduces the risk of overfitting. It is also robust to outliers and can handle both numerical and categorical data. Additionally, RF is easy to use and interpret. It can also provide variable importance, which can help to determine the most important features in a data set. Finally, RF is computationally efficient, since it can handle large data sets with minimal memory and computational resources.

The models identified several demographic, medical, and lifestyle factors that influence the age at the onset of childhood obesity. The demographic factors that were identified to be significant include gender, income and education level of father and mother, and family history of obesity. The significant medical factors include autism, gestational age (in weeks), height, nutrition in the first 6 months, and weight at birth. The significant lifestyle factors include child sleep hours, consanguineous marriage, fast food and sweets, consumption of solid food in the first 6 months, watch TV per day. The lifestyle factors are easy to address through campaigns and policies to increase awareness. Some of the findings from this study are consistent with other similar national and international studies. For example, [29, 33, 34], found that parental obesity [29], low physical activity and a high intake of fast food and sugary drinks were significant predictors of early-onset obesity in children. The findings also agree with other international studies. For example, [24, 48] found early childhood overweight is associated with later childhood obesity, [21, 22] and found income and education level of the father and mother are significant factors. A Denmark study showed that a primary prevention intervention targeting obesity-susceptible, healthy-weight preschool children effectively mitigated fat and weight gain [16].

## Conclusion

Childhood and adolescent obesity is a major global public health threat. Worldwide, 5.6% of girls and 7.8% of boys were obese in 2016 [1]. In the U.S., 17% of children were obese and another 15% were overweight in 2010 [49]. In Saudi Arabia, 9.4% of the children and adolescents population is obese, and 11.2% is overweight [27]. Childhood obesity increases the risk of type II diabetes, cardiovascular disease, metabolic syndrome, and later life obesity, and has adverse effects on pulmonary, musculoskeletal, and psychosocial functioning. To the best of our knowledge, this is one of the first papers that employed Multiple Linear Regression, Random Forest, Decision Tree, and K-Nearest Neighbour models to predict the age at the onset of childhood/adolescent obesity in Saudi Arabia and to identify the most significant factors associated with it. The results revealed that R.F. with *R*^2^ = 98.44, RMSE = 0.44, and MAE = 0.28 outperforms other models followed by MLR, DT, and KNN.

The analysis also shows that early life factors such as birth weight, gestational age, and parental feeding behaviors are associated with childhood and adolescent obesity. The results, from this reasonable size cohort, contribute to the body of literature suggesting that the risk of childhood obesity often starts in early life. Efforts to identify interventions to prevent childhood obesity should focus on the periods of gestation and infancy as obesity risk may be malleable during these periods. This finding can be ustilised to develop strategies to identify children at risk of obesity, enabling early intervention and prevention. Additionally, the findings emphasize the need for increased education on healthy lifestyle habits and nutritional knowledge. Furthermore, the research highlights the importance of public health initiatives and policies to address childhood obesity. Including a larger number of cities in the research would improve both the diversity and sample size which is a limitation of the current study and would provide a more robust prediction model. Furthermore, continued research into the other factors contributing to obesity onset age (e.g., genetic factors, race/ethnicity) is recommended to improve the accuracy of predictive models.
